# Distribution of Potential Harmful Trace Elements and Potential Ecological Risk in the Jiulongchi Wetland of Fanjing Mountain, Southwest China

**DOI:** 10.3390/ijerph17051731

**Published:** 2020-03-06

**Authors:** Weidan Shen, Kangning Xiong, Yang Gao, Mingying Quan, Haijun Peng, Ting Yang, Linfeng He, Kunshan Bao

**Affiliations:** 1 School of Karst Science, Guizhou Normal University/State Key Laboratory Incubation Base for Karst Mountain Ecology Environment of Guizhou Province, Guiyang 550001, China; shenwd1991@163.com (W.S.); xiongkn@163.com (K.X.); 2 School of Geographic Sciences, South China Normal University, Guangzhou 510631, China; temiaquan@gmail.com (M.Q.); yt1295178192@163.com (T.Y.); 3 State Key Laboratory of Environmental Geochemistry, Institute of Geochemistry, Chinese Academy of Sciences, Guiyang 550081, China; penghaijun@mail.gyig.ac.cn; 4 School of Chemistry and Materials Science, Guizhou Normal University, Guiyang 550025, China; helinfeng9889@163.com

**Keywords:** harmful trace elements, alpine wetland, ecological assessment, multivariate analysis, Southwest China

## Abstract

In order to understand the distribution and ecological risk of potential harmful trace elements (PHTEs) in the high altitude areas of the Fanjing Mountain World Natural Heritage Property, 30 surface samples including soil and plants were collected in April, 2019 in the Jiulongchi wetland which lies in the saddle between the New Jinding Peak of Fanjing Mountain and Fenghuang Peak. The contents of 23 major and trace elements were determined, and the pollution characteristics and potential ecological risk of 11 PHTEs (Mn, V, Zn, Cr, Co, Ni, Cu, As, Cd, Sb and Pb) were discussed. The element contents showed significant differences in plant and soil samples. Enrichment factor and single-factor pollution index demonstrated that Mn, Zn, Co, As and Cd in the soil were in a clean state. The potential ecological risk index and pollution load index indicated an overall good ecological condition of Jiulongchi wetland, with a weak pollution degree. Comparisons of relevant studies showed the anthropogenic activities have considerable impacts on the pollution status of PHTEs with significant spatial differences in Fanjing Mountain. Multivariate statistical analysis proved that Pb and Sb were main pollutants of the soil in the Jiulongchi wetland, and the mining and smelting of minerals such as mercury, manganese and lead-zinc ore in the adjacent areas could be the main pollution sources through atmospheric deposition. This study could provide targeted strategies for the environmental protection and management of the Property and give scientific evidence for the pollution prevention in southwest China.

## 1. Introduction

Good environmental conditions are a necessity for human survival and development. With the social development, especially since the industrial expansion in the 1950s, the anthropogenic impacts on natural soil environment have become more and more intense, which has led to serious soil erosion and pollution problems. The potentially harmful trace elements (PHTEs) introduced into soil through human activities can change the original constituents and soil quality, which is harmful to the natural environment and human health. The problem is still getting worse on a global scale [1].

Guizhou Province is rich in mineral resources with a history of thousands of years of mining and smelting activity [2]. The industries in Guizhou Province are based on the enriched resources, taking energy, raw material, machinery and electronics with flue-cured tobacco, cigarettes and wine as predominant outputs [3]. Those industrial production processes have led to industrial problems, especially PHTEs pollution [4,5].

Fanjing Mountain is the main peak of the Wuling Mountains of China, being listed in the World Heritage List in 2018 for its biological diversity. The excellent ecological environment guarantees the outstanding universal value (OUV) in Fanjing Mountain World Natural Heritage Property. However, a study showed that the contents of Cd, Cr, Hg, As and Pb exceeded the national soil quality standard (GB151618-1995) [6]. Another report about the heavy metals in forest soil of Fanjing Mountain also suggested that soil environment had been mainly affected by Cd, Pb and Hg [7]. A ~400-yr history of atmospheric trace elements pollution was reconstructed based on Jiulongchi sediments of Fanjing Mountain and proved that Cd and Pb were mainly from regional nonferrous metals smelting and coal combustion [8]. In the context of the continuously intensified human activities, it is advisable to study the current ecological risk in this alpine wetland ecosystem of the precious World Natural Heritage Property.

This study aimed to clarify the distribution characteristics of main PHTEs in the Jiulongchi wetland area of Fanjing Mountain, reveal the pollution status of the Fanjing Mountain high-altitude representing our study area, and conduct a potential ecological risk assessment. This should provide scientific references and suggestions for the prevention and control of PHTEs, environmental protection and management and ecological activities in the property and even throughout Southwest China.

## 2. Material and Methods

### 2.1. Study Area

**Figure 1:**
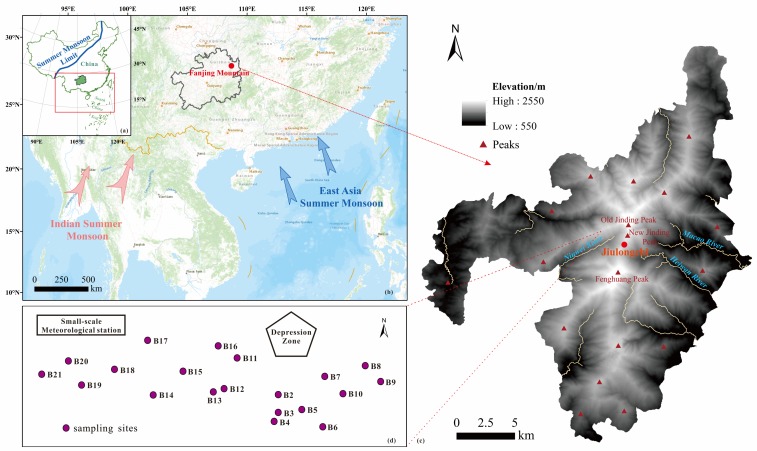
The location of Guizhou Province in China (**a**), Fanjing Mountain in Guizhou Province (**b**), elevation map of Fanjing Mountain (**c**) and the sketch map of sampling sites (**d**).

Guizhou Province lies in the southwest of China (Figure 1a), and Fanjing Mountain (27°53′44″ N, 108°40′48″ E) is located in Tongren City, in northeast Guizhou Province, lying in the transition zone from the Yunnan-Guizhou Plateau to the Western Hunan Hills. The Precambrian base geology is widely distributed and well preserved in this area, which is composed mainly of the Fanjingshan Group and Neoproterozoic Banxi Group. The orogeny of regional collision and erosion gave the birth to Fanjing Mountain with metamorphic rock, and it is significantly different from surrounding karst landforms [9]. The location and high elevation (~2570 m asl) have caused the specific environment: subtropical humid monsoon climate in mountain area with abundant water (average annual rainfall of 1100–2600 mm and more than 20 rivers) and heat (average annual temperature 6–17 °C), an altitudinal range of more than 2000 m and obvious vertical differentiation with five major altitudinal vegetation zones from evergreen broadleaf forest (<1300 m asl) to meadow (>2400 m asl), with abundant biodiversity with many endemic and relict species. The main soil types here are yellow soil and yellow-brown soil, and Fanjing Mountain is also the watershed of the Wujiang River Basin and Yuanjiang River Basin, and is affected by both the India Summer Monsoon and East Asian Summer Monsoon (Figure 1b) [10,11].

The sampling site, the Jiulongchi wetland, lies on the saddle between anticline dome of Jinding Peak and Fenghuang Peak (Figure 1c). It is about 2 km away from the New Jinding Peak. There are *Polytrichum commune*, deciduous broad-leaved dwarf forests and mountain dark coppice soil, surrounded by mountains on three sides. It was once a cirque lake. The Jiulongchi wetland belongs to the core zone of the property site where any construction and activities are forbidden except for authorized scientific research activities in the zone to protect the natural environment, and is therefore less affected by human activities. The sedimentary environment has been stable and continuous since Holocene [10]. As a water-land transitional natural ecosystem, it is sensitive to regional environment changes.

### 2.2. Sampling

A total of 20 soil samples (the 0–2 cm soil) and 10 plant samples (moss and grass, but mainly moss) were collected from the Jiulongchi wetland in April, 2019. The locations of the sampling sites were recorded by a portable Global Positioning System instrument (Gamin GPS 62SC, Garmin International, Olathe, KS, USA, Table 1). The samples were sealed into labeled zip-lock polyethylene plastic bags, and brought back to laboratory, where they were stored at low temperature (4 °C).

### 2.3. Sample Analysis

All the samples were dehydrated in an oven at 45 °C, and the minor sand particles and root residues of soil samples were picked out with plastic tweezers. Then, every sample was ground with an agate mortar until there is no obvious grains to the touch.

#### 2.3.1. Physicochemical Analysis

The organic matter content of the samples was measured by the loss-on-ignition (LOI) method. Clean crucibles were numbered with the sample code, and heated in the oven at 85 °C for 2 h to constant weight (recorded as m_0_). Ground samples were added into the corresponding crucibles, and weighted (recorded as m_1_). The mass of samples can be calculated by the Δ of m_1_ and m_0_. These crucibles with samples were calcined in the muffle furnace (Jinghong, Hebei Hinstr Electronic Technology Ltd., Baoding city, China) at 550 °C and at 950 °C for 4 h and 1 h, respectively. After cooling, the weights were recorded as m_3_ and m_4_ respectively. Lost masses of the samples at 550 °C and at 950 °C are “m_1_-m_3_” and “m_3_-m_4_” respectively. The LOI at 550 °C is the percentage of organic matter (OM) in samples. The content of total organic carbon (TOC) is estimated from half of the OM. The LOI at 950 °C represents the percentage of total inorganic carbon (TIC). The sum of TOC and TIC is the content of total carbon (TC) [12].

#### 2.3.2. Trace and Major Elements Analysis

Geochemical analysis of the wetland soil and plant samples followed a digestion procedure previously established by the State Key Laboratory of Lake and Environment, Nanjing Institute of Geography & Limnology, Chinese Academy of Sciences [13,14]. Ground homogeneous samples (200 mg) were accurately weighed into the TFM-PTFE lined vessel of a stainless steel pressure digestion system (DAB-2, Berghof, Berchtesgaden, Germany). We added 0.5 mL H_2_O_2_ (Guaranteed Reagent, Shanghai, China) and 2.5 mL high-purity HNO_3_ which were obtained by sub-boiling distillation of the analytical grade reagent in an I.R. distiller (BSB-939, Berghof). Then the PTFE vessels were closed and heated at 200–200 °C for 3 h. After cooling, 1 mL HF (sub-boiling distillation high-purity) and 0.5 mL HClO_4_ (Guaranteed Reagent, Tianjin, China) were added to the PTFE vessels, and they were heated until no more white smoke appeared from the liner. High-purity HNO_3_ (0.5 mL) and a small volume of deionized water (>18 MΩ·cm) were added after cooling again, then heated again at 150–180 °C for 5 min and cooled to ambient temperature for more than 2 h. Next, the digested samples were transferred into a 50 mL centrifuge tube along with the washings from the TFM-PTFE liner. Lastly, the sample solutions were brought up to a final volume of 25 mL with deionized water for the elemental analysis by atomic emission spectroscopy with inductively coupled plasma (ICP-AES Prodigy 7, Teledune Leeman Lab, Hudson, NH, USA) and quadrupole inductively coupled plasma mass spectroscopy (Q-ICP-MS 7700x, Agilent Technologies, Santa Clara, CA, USA). Four parallel samples were set randomly to ensure the accuracy of the determination with replicate analyses (4 parallel samples from sample 1, 12, 27 and 16 respectively, shown in Table 2). The elements’ limit of detection (LOD) was also shown in Table 2.

### 2.4. Parameter Calculations

#### 2.4.1. Enrichment Factor

The enrichment factor (EF) was first proposed to determine atmospheric particulate concentrations and sources of trace metals at the South Pole [15]. By comparing the measured value and natural background value of the target element and reference element, the pollution degree of target element and sources can be calculated as:

(1) $$\mathrm{EF}=\frac{{{(C}_{i}/S_{x})}_{\mathrm{sample}}}{{{(C}_{i}/S_{x})}_{\mathrm{Guizhou}}},$$

where EF stands for the enrichment factor, (C_i_/S_x_)_sample_ stands for the ratio of measured value (mg·kg^−1^) of element *i* to measured value (mg·kg^−1^) of reference element *x* in the sample, (C_i_/S_x_)_Guizhou_ stands for the ratio of background value (mg·kg^−1^) of element *i* to the background value (mg·kg^−1^) of reference element *x* in Guizhou soil.

The reference elements are generally conservative elements that are stable and resistant to weathering under natural conditions, such as Al [16], Fe [17] and Ti [18]. In order to avoid the effects of biogeochemical processes, the average value of EF_Al_, EF_Fe_ and EF_Ti_ was used as the comprehensive EF value for evaluating pollution elements in samples. EF value <1 indicates a clean state, 1–2 indicates a light pollution, and >2 indicates serious pollution.

#### 2.4.2. Single-Factor Pollution Index

As a widely used method in evaluating the PHTEs pollution degree in soil, the single-factor pollution index (P_i_) was used as the assessment criterion (Equation (2)):

(2) $$P_{i}=\frac{C_{i}}{S_{i}},$$

where C_i_ stands for the content measured value (mg·kg^−1^) of element *i* in the sample, and S_i_ stands for the corresponding content reference value (mg·kg^−1^). The elements’ background values of A layer soil in Guizhou Province [19] was used as the reference. Value <1 indicates a clean state, 1–2 indicates a light pollution, and >2 indicates serious pollution.

#### 2.4.3. Pollution Load Index

Tomlinson proposed the pollution load index (PLI) based on the single-factor pollution index to evaluate the heavy-metal pollution from industries in estuaries [20]. Multi-elements pollution can be evaluated by PLI, which has been used widely to reflect the composition of the pollution elements in the target areas (Equations (3) and (4)):

(3) $${\mathrm{PLI}}_{\mathrm{site}}=\sqrt[{n}]{P_{i\;1}{\times P}_{i\;2}{\times P}_{i\;3}{\times \ldots \times P}_{i\;n}},$$

(4) $${\mathrm{PLI}}_{\mathrm{zone}}=\sqrt[{m}]{{\mathrm{PLI}}_{1}{\times \mathrm{PLI}}_{2}{\times \mathrm{PLI}}_{3}{\;\times \ldots \times \;\mathrm{PLI}}_{m}},$$

where P_i_ is the single-factor index; n and m refer to the number of evaluated elements (11, Mn, V, Zn, Cr, Co, Ni, Cu, As, Cd, Sb and Pb) and the number of sampling sites (20) respectively. A PLI value not exceeding 0.7 represents the clean state. The value between 0.7–1 means a slight pollution level, 1–2 means the mild state, and >2 means the serious state.

#### 2.4.4. Potential Ecological Risk Index

This ecological risk method was proposed by Swedish scientist Håkanson in 1980 (Equation (5)), which comprehensively considers the concentration, type, toxicity and environmental sensitivity of pollution elements [21]:

(5) $$\mathrm{RI}\;=\sum E_{r}^{i}=\sum T_{r}^{i}{\times \;C}_{f}^{i}=\sum T_{r}^{i}\times \frac{C_{i}}{S_{i}},$$

where RI stands for the potential ecological risk index, $E_{r}^{i}$ stands for the potential ecological risk factor of element i, $T_{r}^{i}$ stands for the toxic response factor of element i, $C_{f}^{i}$ stands for the factor of contamination of element *i*, and its calculation method is the same as the single-factor pollution index. The $T_{r}^{i}$ values of 7 pollution elements were given by Håkanson, and others were referenced to related studies [22,23,24]: Mn = Zn = 1, V = Cr = 2, Co = Ni = Cu = Pb = 5, Sb = 7, As = 10, Cd = 30. $E_{r}^{i}$ ≤ 40 and RI ≤ 150 represent the low ecological risk.

### 2.5. Statistical Analysis

To determine the causes and sources of the pollution in study area, multivariate statistical analysis of pollution elements was performed, including principal component analysis, cluster analysis, and correlation analysis. These methods can achieve the dimension reduction and simplification of complicated and related multi-indexes, and merging relatively homogeneous factors [25]. All above analyses were done using Excel 2016 (Microsoft Co., Redmond, WA, USA), Origin 8.0 (OriginLab, Northampton, MA, USA) and IBM SPSS 22.0 (IBM Co., Chicago, IL, USA), and the spatial distributions of the 11 PHTEs were done by ArcGIS 10.2 (Environmental Systems Research Institute, Inc., Redlands, CA, USA) with simple kriging.

## 3. Results

### 3.1. The Loss-on-Ignition of 30 Samples

**Figure 2:**
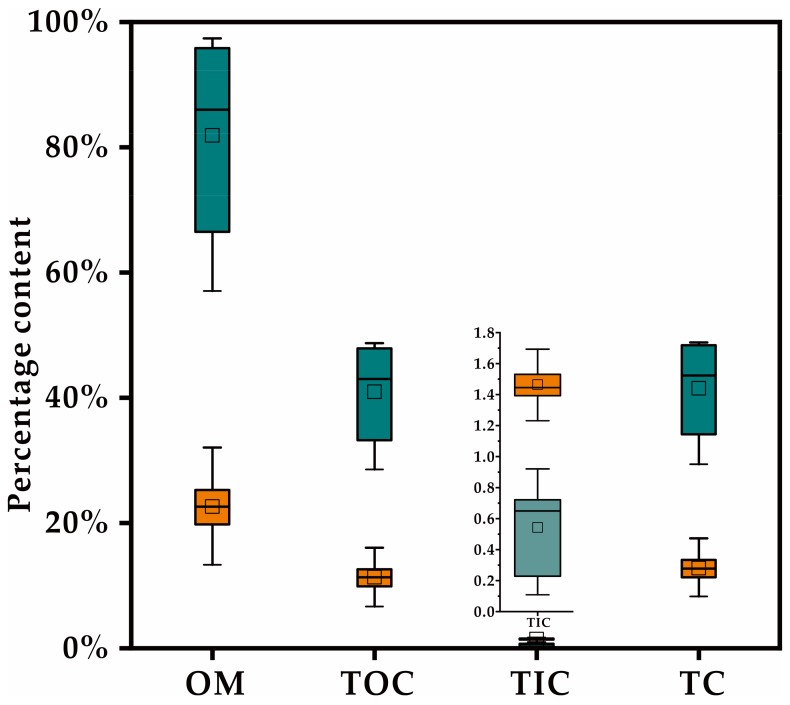
The boxplot of OM and carbon percentage content in soil samples (orange) and plant samples (cyan). The bottom, top and medium band of the box are the 25th, 75th and 50th percentiles. The horizontal line above and below the box are the maximum and minimum value, and the square in the box is the mean respectively.

The percentage contents of OM, TOC, TIC and TC for the 20 soil samples were 22.62%, 11.31%, 1.46% and 12.78%, and 81.89%, 40.95%, 0.54% and 41.49% for the 10 plant samples, respectively. The means of OM and TOC in soil samples are relatively low (less than 35% and 20%, respectively). For plant samples, the contents of OM, TOC and TC are far higher than those for soil samples. TIC contents in all samples are very low (0.11–1.69%) (Figure 2).

### 3.2. The Descriptive Statistics and Spatial Distributions of Element Contents

**Figure 3:**
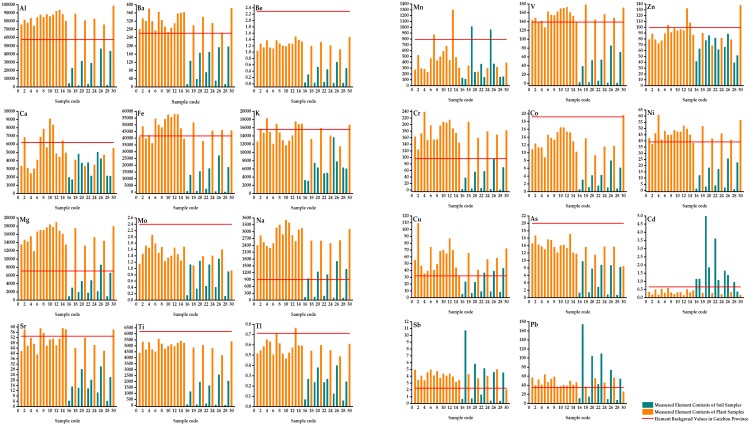
The contents and corresponding background values of 23 elements in 30 samples from Jiulongchi wetland (mg·kg^−1^).

**Figure 4:**
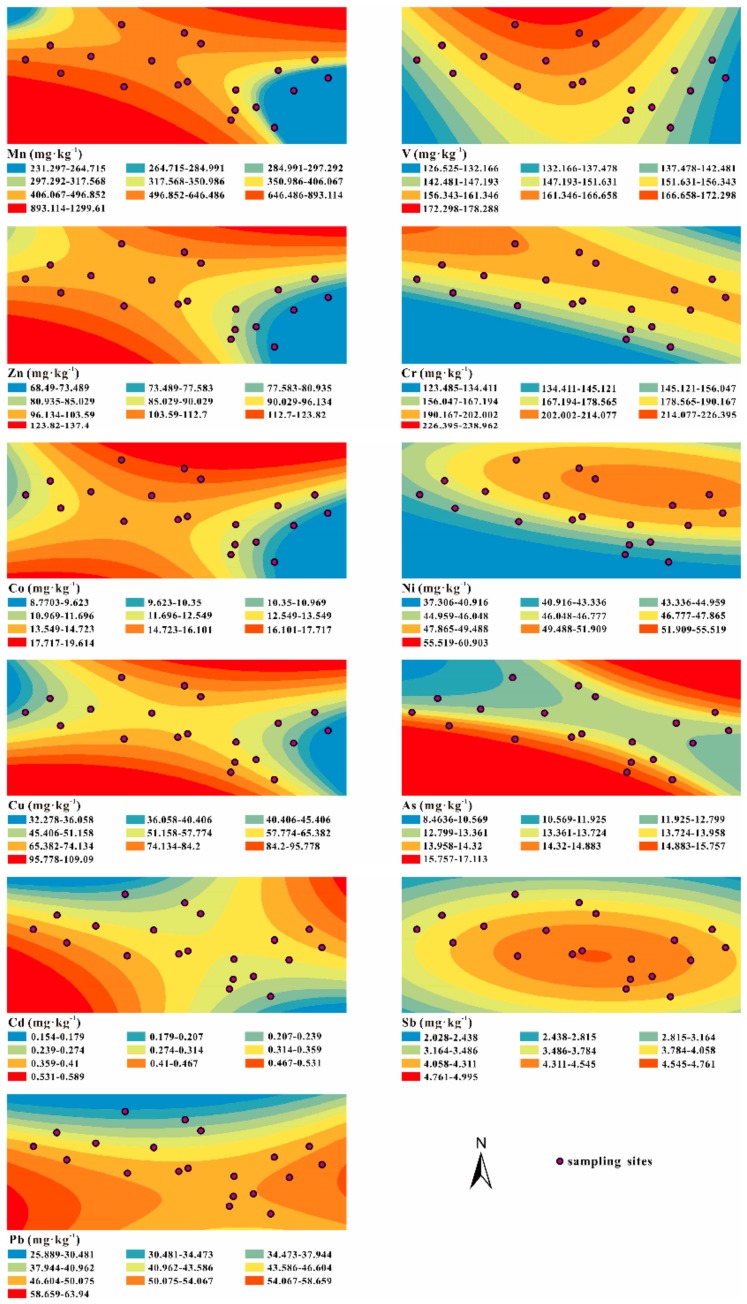
Spatial distributions of 11 PHTEs in 20 surface soil samples from Jiulongchi wetland of Fanjing Mountain, southwest China.

Considering the types of industries in the Tongren area, 11 PHTEs, Mn, V, Zn, Cr, Co, Ni, Cu, As, Cd, Sb and Pb, were selected to analyze the pollution status in the study area. Their contents and the descriptive statistics analyses of 23 determined elements are shown in Table 3 and Figure 3. The analysis of each element is described from two aspects in the Table: soil and plant samples. The plants are mainly moss (*Polytrichum commune*). The spatial distributions of 11 PHTEs in 20 soil samples are mapped in Figure 4.

### 3.3. The Pollution Evaluations

The results of above four evaluation methods for 11 PHTEs from the Jiulongchi wetland soils are shown in Table 4. The EFs and P_i_s show that Mn, Zn, Co, As and Cd are in a clean state, and V, Cr, Ni, Cu, Sb and Pb are in a light pollution state. The potential ecological risk index and pollution load index suggest that the Jiulongchi wetland is currently under slight pollution and at low risk. 

**Figure 5:**
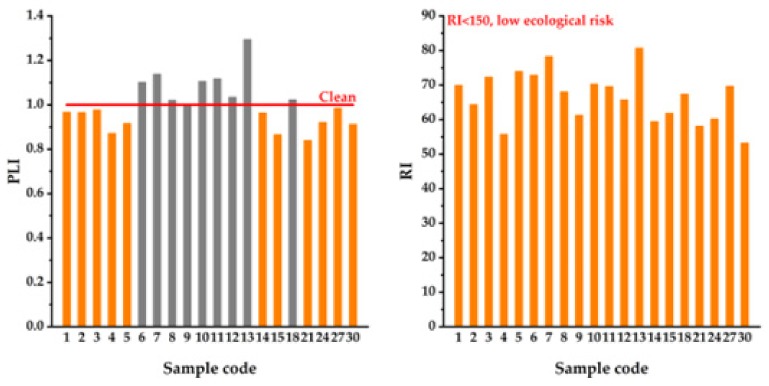
The results of PLI and RI evaluations from 20 surface soils for the sampling sites. The gray columns indicate nine sites in the center of Jiulongchi wetland are in a light pollution state which is in accordance with the spatial distribution of 11 PHTEs.

In order to better understand the environment state of the Jiulongchi wetland, the soil samples of 20 sampling sites were also evaluated by the PLI and RI indexes (Figure 5). As shown by PLI, the pollution level of sample 6 to 13, and 18 (the gray columns) indicates mild pollution. The RI shows all sites are under low ecological risk. The evaluation results of each sampling sites are consistent with those of the entire Jiulongchi wetland zone.

### 3.4. The Correlation and Principal Component Analysis

The Pearson correlation results are summarized in Table 5. Significantly positive correlations are observed between most of the PHTEs. However, Mn is only correlated with Zn, and Pb is only significantly correlated with Sb. There are significant negative correlations between Cd and the elements V, Cr, Co, Ni, Cu and As. It worth noting that As is significantly positively correlated with Sb (Table 5).

**Figure 6:**
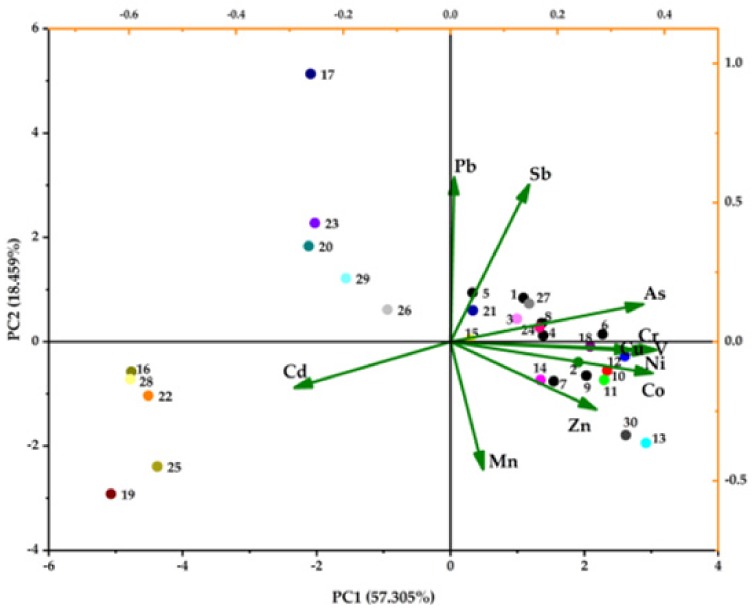
The PCA biplot of 11 PHTEs and 30 samples in the Jiulongchi wetland. The different color dots and the numbers next to them represent the collected 30 samples and corresponding codes.

Due to the close relationship of the trace elements between plants and soil, principal component analysis (PCA) was performed on the 11 PHTEs from 30 samples (Figure 6). The value of Kaiser-Meyer-Olkin is 0.760 and the Bartlett value is 0. Three components (PC1-PC3) can be extracted (Eigenvalue > 1), which accounts for 90.918% of the total variance. For 11 PHTEs, at least 76.2% or more of their variance is explained by the three components. PC1 (Eigenvalue = 6.303) contains a positive loading (0.853–0.978) of V, Ni, Cr, Co, As and Cu and a negative loading of Cd (−0.830), and accounts for a large proportion of the total variance (57.305%). PC2 (Eigenvalue = 2.031) contains a positive loading of Pb and Sb (0.980 and 0.940) and accounts for 18.459% of the total variance. PC3 (Eigenvalue = 1.667) contains a positive loading of Mn and Zn (0.900 and 0.722) and accounts for 15.154% of the total variance. In addition, 30 samples are divided into 3 categories as the sample texts. The samples in second quadrant are root and soil, third quadrant are stem and leaf, and others are soil (Figure 6).

## 4. Discussions

### 4.1. The Spatial Distributions of PHTEs in Jiulongchi Wetland Soil

Wilding proposed that a coefficient of variation (CV) value under 15%, between 15% and 36%, and over 36%, should be classified as low, moderate, and high variability, respectively [28]. According to the Wilding analysis criterion, V, Ni and As are of low variability and distributed evenly in the Jiulongchi wetland. Zn, Cr, Co, Cu, Sb and Pb are of moderate variability, while Mn and Cd show high variability in the Jiulongchi wetland soil (Table 3). The result indicates the distribution of the 11 elements is not homogeneous. As, Cu, Co, Cd, Mn and Zn have a similar distribution pattern, with high-content values occurring in the NE and SW directions of the Jiulongchi wetland. Both V and Ni have relatively high content values in the north (near the depression zone), and the Sb content is decreasing from the center to the surroundings. Cr is highly concentrated in a small area in the NW (near the small-scale meteorological station) and shows a low-content in the SW and NE. High contents occur in the west and east for Pb (Figure 4).

Relatively high contents of all PHTEs exist in the lower area near the depression zone, which might be related with the wetland water flow and their mobility in soil. In addition, the Pearson correlation analysis on the contents of 11 PHTEs and OM and TIC in 20 soil samples showed significant correlations between OM and V (*r* = −0.468, *p <* 0.05, 2-tailed), Ni (*r* = −0.516, *p* < 0.05, 2-tailed), Zn (*r* = −0.647, *p* < 0.01, 2-tailed), Co (*r* = −0.615, *p* < 0.01, 2-tailed), Sb (*r* = 0.753, *p* < 0.01, 2-tailed) and Pb (*r* = 0.693, *p* < 0.01, 2-tailed). Ni has a significant positive correlation with TIC (*r* = 0.483, *p* < 0.05, 2-tailed). These correlations suggest a different influence of soil textures on the trace element distribution. The Sb and Pb in soil is more likely absorbed by OM [29] and the others would be probably controlled by the soil forming process.

Our results are comparable with two reports about heavy metals distribution in Fanjing Mountain Nature Reserve soil [6,7]. For example, the contents of As and Pb in Jiulongchi wetland soil are 13.6 mg·kg^−1^ and 46.2 mg·kg^−1^, which are consistent with Lu’s study (8.6 mg·kg^−1^ and 40.2 mg·kg^−1^) [6] and another report (11.4 mg·kg^−1^ and 54.3 mg·kg^−1^) [7]. Comparing our results with other related studies (Table 3), it can be found that the contents of As, Cr, Cu, Cd, Pb, Sb, Ti, V, and Zn in Jiulongchi wetland soil are higher than the Earth’s Upper Continental Crust (UCC) [27] and the background values of China soil elements (BK China) [19]. In addition, comparing with the BK Guizhou [19], BK Hunan [19] and BK Sichuan [19], the contents of Cu, Cr, Ni, Pb and Sb in Jiulongchi wetland soil are higher. The contents of Zn, Cr, Ni, Cu, As, Cd and Pb in Jiulongchi wetland soil are also higher than those in Giant Panda National Park, Qinling Mountains [26]. Therefore, it can be deduced that human activities have considerable impacts on the environment quality in the Fanjing Mountain area.

### 4.2. The Soil Pollution Evaluation of Jiulongchi

The evaluation results of EF and P_i_ show a clean state for Mn, Zn, Co, As and Cd in the Jiulonogchi wetland soil. However, some studies have shown Cd is a main pollutant in Guizhou Province [6,7,8,30]. The reason for the difference might be the high altitude of Jiulongchi (~2035.5 m asl) and a non-karst landform of Fanjing Mountain. The Cd pollution in Guizhou Province is mainly due to carbonate rock weathering [31,32,33]. All the PLI values are less than 2, ranging from 0.84 to 1.29. For Jiulongchi wetland, the PLI_zone_ value is 0.99, indicating a slight pollution state. The value of $E_{r}^{i}$ for 11 PHTEs ranged from 0.58 (Mn) to 14.61 (Cd) with an average value of 5.73, which are far below the critical value, 40. The RI value of Jiulongchi wetland is 66.59, which is also much smaller than the critical value, 150. Both PLI and RI show a good consistency. The results indicate a light pollution degree in Jiulongchi wetland (mainly close to the central zone, Figure 5), and the soil environmental quality is still good, in spite of the anthropogenic impacts.

### 4.3. The Sources Analysis of PHTEs in Jiulongchi Wetland

In addition to natural lithogenic source, those pollution elements also come from human activities, mainly industry and agriculture. The three evaluation parameters (EF, P_i_ and RI) show Co, As, and Cd are in a clean state, and they are included in PC1. Thus, PC1 is considered to be mainly from natural sources. This is consistent with some previous studies about Cu and Cr in soil dominated by the region’s geology characters [34,35]. There is a relatively high background of trace metals in Guizhou and rich mineral resources in Tongren Region, Guizhou Province. This would be the main reason for most of PHTEs showing natural contribution, though the evaluation indices show their higher pollution potential. As typical pollution elements, according to the correlation analysis in Table 5, Pb and Sb are with a significantly positive correlation (*r* = 0.891, *p* < 0.01) in PC2, which is considered they come from anthropogenic sources. Part of As also belongs to this component. There are lots of industrial activities, such as exploitation and smelting of mercury ore and lead-zinc ore, around the study area. In addition, chemical production, automobile exhaust, and industrial and domestic coal combustion are also extensive in the neighboring metropolis like Guiyang and Chengdu. Both Mn and Zn in PC3 might be considered as redox effect in the wetland soil. Mn and Fe oxides, as common components, are distributed in soil widely, and many trace elements can be absorbed and immobilized by them. Zn behavior is governed by redox chemistry of Fe and Mn oxides under oxidizing conditions [36,37].

### 4.4. Management Significance for Fanjing Mountain World Natural Heritage Property

The Jiulongchi wetland is located at high altitude and in a remote location, and thus the current environmental quality is still good. However, it is worth noting that most of PHTEs values are higher than the regional background values, showing the possible anthropogenic impacts. There is still a certain degree of pollution risk in the Fanjing Mountain region, especially with the accelerated regional industrialization process. Therefore, the comprehensive monitoring for ecological environment quality should be emphasized in the future protection and management work. The smelting and mining of nearby minerals, such as mercury ore, manganese ore, lead-zinc ore, should be reasonably controlled. At the same time, publicity and environment education can be enhanced for supporting of ecological civilization construction (a modern environment protection concept of Chinese government) and environmental protection. The regional network can be implemented to jointly monitor the pollution sources to protect the ecosystem of Fanjing Mountain, the precious World Natural Heritage Property, from being negatively affected.

## 5. Conclusions

The pollution patterns of 11 PHTEs were investigated through geochemical analysis of soil and plant samples from the Jiulongchi wetland of Fanjing Mountain in Southwest China. The multi-indexes evaluations of 11 PHTEs in soil show that V, Ni, Cr, Co, As, Cu and Cd are mainly from natural sources. Mn and Zn are related with the redox effect in the wetland soil. Both Sb and Pb are main pollutants from anthropogenic sources and cause light pollution. The potential ecological risk index (66.59) and pollution load index (0.99) indicate a slight pollution degree for the Jiulongchi wetland with a low ecological risk. The current environmental quality of high-altitude alpine ecosystem in Fanjing Mountain World Natural Heritage Property is still good. However, considering the potential pollution from the industrial production in the adjacent area, more attention needs to be paid for wise management in the World Heritage Property.

## Figures and Tables

**Table 1:** The details of sampling sites and samples from the Jiulongchi wetland of Fanjing Mountain.

Sampling Site ^1^	Sample Code	Longitude (E)	Latitude (N)	Elevation (m asl)	Sample Type
B1	0	108°41′33.35″	27°53′57.79″	2037	Water (Ph = 7.18, EC = 40 µS/cm ^2^)
B2	18	108°41′33.36″	27°53′57.53″	2035	Soil
	16				Grass and Moss
	17				Root and Soil
B3	1	108°41′33.35″	27°53′57.29″	2033	Soil
B4	2	108°41′33.30″	27°53′57.17″	2036	Soil
B5	21	108°41′33.61″	27°53′57.32″	2037	Soil
	19				Grass and Moss
	20				Root and Soil
B6	24	108°41′33.83″	27°53′57.08″	2035	Soil
	22				Grass and Moss
	23				Root and Soil
B7	27	108°41′33.88″	27°53′57.76″	2038	Soil
	25				Grass and Moss
	26				Root and Soil
B8	3	108°41′34.33″	27°53′57.89″	2035	Soil
B9	4	108°41′34.49″	27°53′57.67″	2037	Soil
B10	5	108°41′34.07″	27°53′57.52″	2037	Soil
B11	30	108°41′32.93″	27°53′58.04″	2037	Soil
	28				Grass and Moss
	29				Root and Soil
B12	6	108°41′32.77″	27°53′57.63″	2035	Soil
B13	7	108°41′32.65″	27°53′57.59″	2036	Soil
B14	8	108°41′31.99″	27°53′57.57″	2035	Soil
B15	9	108°41′32.33″	27°53′57.88″	2037	Soil
B16	10	108°41′32.73″	27°53′58.21″	2034	Soil
B17	11	108°41′31.96″	27°53′58.31″	2035	Soil
B18	12	108°41′31.58″	27°53′57.93″	2035	Soil
B19	13	108°41′31.21″	27°53′57.73″	2034	Soil
B20	14	108°41′31.08″	27°53′58.06″	2034	Soil
B21	15	108°41′30.78″	27°53′57.89″	2035	Soil

^1^ Those sites are shown in the sketch map of Jiulongchi wetland in Figure 1d. ^2^ Measured on site by a pH-100 instrument (Lichenkeyi Co., Ltd., Shanghai, China) and a DDB-12L instrument (Qiwei Instrument Co., Ltd., Hangzhou, China).

**Table 2:** Replicate analyses of element contents (mg·kg^−1^) with 4 parallel samples expressed as mean ± standard deviation and the 23 major and trace elements’ limit of detection (LOD).

Element	Parallel Sample Code				LOD	
	1	12	27	16	Soil	Plant
**Al**	75,799.84 ± 3.39	92,115.1 ± 48.9	75,754 ± 46.73	4224 ± 24.41	20	10
**Ba**	280.62 ± 0.35	309.71 ± 0.22	264.08 ± 0.52	12.74 ± 0.1	0.5	0.5
**Be**	1.05 ± 0.01	1.27 ± 0.02	1.06 ± 0.03	0.04 ± 0	0.2	0.02
**Ca**	3342.53 ± 3.68	4848.17 ± 15	4687.98 ± 16.33	1979.63 ± 2.13	5	5
**Fe**	41,139.47 ± 135.9	57,927.79 ± 7.96	46,144.89 ± 153.85	711.46 ± 10.06	5	5
**K**	12,575.85 ± 44.04	14,090.26 ± 0.22	11,465.74 ± 35.26	3256.76 ± 3.31	80	40
**Mg**	13,547.31 ± 37.51	18,960.17 ± 2.53	14,442.77 ± 17.45	895.63 ± 4.47	2	5
**Na**	2401.19 ± 0.44	2827.95 ± 7.47	2610.69 ± 0.31	124.34 ± 0.79	20	5
**Sr**	41.72 ± 0.02	45.92 ± 0.17	41.98 ± 0.1	4.32 ± 0.02	0.2	0.2
**Ti**	4390.89 ± 6.5	4935.2 ± 13.81	4203.69 ± 20.64	63.48 ± 1.36	1	1
**Mo**	1.2 ± 0.07	1.69 ± 0.05	1.57 ± 0.03	0.15 ± 0	0.05	0.01
**Tl**	0.51 ± 0	0.58 ± 0.01	0.49 ± 0.01	0.07 ± 0	0.02	0.005
**Mn**	273.61 ± 0.03	437.64 ± 1.50	316.29 ± 1.04	132.42 ± 0.58	0.5	0.2
**V**	143.7 ± 1.48	174.64 ± 3.02	149.23 ± 1.5	2.51 ± 0.04	2	0.01
**Zn**	78.76 ± 0.43	95.04 ± 0.4	79.56 ± 0.82	41.71 ± 0.18	2	0.05
**Cr**	164.74 ± 1.91	215.45 ± 1.61	167.47 ± 1.66	2.44 ± 0.01	0.1	0.01
**Co**	10.91 ± 0.07	15.48 ± 0.1	11.75 ± 0	0.58 ± 0.01	0.01	0.005
**Ni**	42.28 ± 0.11	52.22 ± 0.01	40.79 ± 0.13	1.33 ± 0	0.05	0.01
**Cu**	55.54 ± 0.88	76.85 ± 14.09	57.83 ± 0.35	5.26 ± 0.04	0.02	0.01
**As**	14.61 ± 0.02	13.75 ± 0.58	13.39 ± 0.42	1.33 ± 0.04	0.1	0.01
**Cd**	0.35 ± 0.01	0.16 ± 0.01	0.34 ± 0.02	1.14 ± 0	0.01	0.005
**Sb**	5 ± 0.07	4.38 ± 0.02	4.95 ± 0.07	0.65 ± 0.01	0.05	0.005
**Pb**	57.27 ± 0.39	39.34 ± 0.21	56.74 ± 0.08	11.32 ± 0.13	0.01	0.005

**Table 3:** The descriptive statistical analysis of 23 element contents (mg·kg^−1^) in the Jiulongchi wetland and the comparison with related studies.

Element	Soil Samples			Plant Samples			Comparison with Related Studies							
	Mean	SD	CV	Mean	SD	CV	FJM [6]	FJM [7]	Qinling Mts. [26]	BK Guizhou [19]	BK Hunan [19]	BK Sichuan [19]	BK China [19]	UCC [27]
**V**	154.67	13.81	0.09	31.33	32.62	1.04	/	/	/	138.8	105.4	96	82.4	98
**Zn**	92.32	17.74	0.19	65.69	17.76	0.27	39.6	/	91.41	99.5	94.4	86.5	74.2	65
**Cr**	179.37	28.02	0.16	32.95	34.51	1.05	51.3	46.35	43.96	95.9	71.4	79	61	126
**Co**	13.21	2.71	0.21	3.07	2.53	0.82	/	/	/	19.2	14.6	17.6	12.7	24
**Ni**	46.53	5.88	0.13	10.71	9.54	0.89	/	/	25.88	39.1	31.9	32.6	26.9	56
**Cu**	58.84	19.11	0.32	20.27	14.8	0.73	13.6	17.26	22.16	32	27.3	31.1	22.6	25
**As**	13.64	1.93	0.14	5.04	3.89	0.77	8.61	11.36	5.76	20	15.7	10.4	11.2	1.7
**Cd**	0.32	0.13	0.4	1.81	1.41	0.78	1.29	1.35	0.24	0.659	0.126	0.079	0.097	0.1
**Sb**	4.01	0.72	0.18	3.42	3.38	0.99	/	/	/	2.24	1.87	1.28	1.21	0.3
**Pb**	46.21	9.58	0.21	60.05	55.54	0.92	40.2	54.28	18.97	35.2	29.7	30.9	26	14.8
**Al**	84,682.56	6339.79	0.07	18,591.49	18,231.61	0.98	/	/	/	57,600	85,500	62,600	66,200	7.96%
**Ba**	321.48	37.78	0.12	101.54	76.44	0.75	/	/	/	261	383	474	469	584
**Be**	1.26	0.12	0.1	0.26	0.26	0.99	/	/	/	2.29	2.19	2.18	1.95	2.4
**Ca**	5119.79	1910.75	0.37	3168.51	1282.43	0.4	/	/	/	6200	1300	11,300	15,400	3.85%
**Fe**	47,859.66	6949.18	0.15	9594.22	9858.04	1.03	23554	/	/	41,700	39,600	33,000	29,400	4.32%
**K**	14,720.27	2085.48	0.14	6368.08	3002.09	0.47	/	/	/	15,600	18,600	20,200	18,600	2.14%
**Mg**	15,897.03	1980.79	0.12	3509.96	2567.65	0.73	/	/	/	7100	4000	8500	7800	2.20%
**Mn**	462.38	254.68	0.55	363.72	342.11	0.94	262	/	696.14	794	459	657	583	716
**Na**	2807.48	369.82	0.13	696.11	632.11	0.91	/	/	/	900	1700	8500	10,200	2.36%
**Sr**	50.07	6.28	0.13	16.23	8.9	0.55	/	/	/	53	44	120	167	333
**Ti**	4969.86	366.16	0.07	974.08	1015.27	1.04	/	/	/	6200	5200	4000	3800	4010
**Mo**	1.47	0.27	0.18	0.72	0.47	0.65	0.986	/	/	2.4	1.4	1	2	1.1
**Tl**	0.58	0.07	0.13	0.23	0.12	0.51	/	/	/	0.712	0.61	0.548	0.62	0.52

Note: The background value of Chongqing city was included in Sichuan Province; the number of soil samples and plant samples are 20 and 10 respectively. SD, Standard deviation; CV, Coefficient of Variation; FJM, Fanjing Mountain; UCC, Earth’s Upper Continental Crust; BK, Background Value.

**Table 4:** The results of different evaluation methods for 11 PHTEs from 20 soil samples.

Parameter	Mn	V	Zn	Cr	Co	Ni	Cu	As	Cd	Sb	Pb
EF	0.53	1.05	0.87	1.76	0.64	1.12	1.71	0.64	0.46	1.70	1.25
P_i_	0.58	1.11	0.93	1.87	0.69	1.19	1.84	0.68	0.49	1.79	1.31
$E_{r}^{i}$	0.58	2.23	0.93	3.74	3.44	5.95	9.19	6.82	14.61	12.53	6.56
RI	66.59										
PLI_site_	0.84–1.29										
PLI_zone_	0.99										

**Table 5:** Pearson correlation analysis for 11 PHTEs in 30 samples.

Element	Mn	V	Zn	Cr	Co	Ni	Cu	As	Cd	Sb	Pb
**Mn**	1										
**V**	0.127	1									
**Zn**	**0.546**	**0.665**	1								
**Cr**	0.108	**0.968**	**0.606**	1							
**Co**	0.235	**0.962**	**0.772**	**0.928**	1						
**Ni**	0.103	**0.974**	**0.655**	**0.986**	**0.941**	1					
**Cu**	0.152	**0.834**	**0.589**	**0.759**	**0.849**	**0.769**	1				
**As**	0.109	**0.902**	**0.534**	**0.866**	**0.817**	**0.871**	**0.794**	1			
**Cd**	0.248	**−0.734**	−0.214	**−0.707**	**−0.675**	**−0.715**	**−0.639**	**−0.688**	1		
**Sb**	−0.292	0.308	0.083	0.280	0.223	0.282	0.290	**0.545**	−0.340	1	
**Pb**	−0.354	−0.058	−0.073	−0.086	−0.130	−0.063	−0.048	0.221	−0.044	**0.891**	1

Bold numbers: Correlation is significant at the 0.01 level (2-tailed).
